# Human Vascular Pericytes and Cytomegalovirus Pathobiology

**DOI:** 10.3390/ijms20061456

**Published:** 2019-03-22

**Authors:** Donald J. Alcendor

**Affiliations:** Center for AIDS Health Disparities Research, Department of Microbiology, Immunology, and Physiology, School of Medicine, Meharry Medical College, 1005 Dr. D.B. Todd Jr. Blvd., Hubbard Hospital, 5th Floor, Rm. 5025, Nashville, TN 37208, USA; dalcendor@mmc.edu

**Keywords:** HCMV, cytomegalovirus, pericyte, brain, ocular, renal, placenta, inflammation, endothelial, vascular

## Abstract

Pericytes are multipotent cells of the vascular system with cytoplasmic extensions proximal to endothelial cells that occur along the abluminal surface of the endothelium. The interactions between endothelial cells and pericytes are essential for proper microvascular formation, development, stabilization, and maintenance. Pericytes are essential for the regulation of paracellular flow between cells, transendothelial fluid transport, angiogenesis, and vascular immunosurveillance. They also influence the chemical composition of the surrounding microenvironment to protect endothelial cells from potential harm. Dysregulation or loss of pericyte function can result in microvascular instability and pathological consequences. Human pericytes have been shown to be targets for human cytomegalovirus (HCMV) infection and lytic replication that likely contribute to vascular inflammation. This review focuses on human vascular pericytes and their permissiveness for HCMV infection. It also discusses their implication in pathogenesis in the blood–brain barrier (BBB), the inner blood–retinal barrier (IBRB), the placenta–blood barrier, and the renal glomerulus as well as their potential role in subclinical vascular disease.

## 1. Introduction

Pericytes, also known as Roget cells or mural cells, were first described by Eberth and Rouget in the 1870s. The name “pericyte” was introduced by Zimmermann in 1923 [1,2,3,4]. Pericytes are described as cells abluminal to endothelial cells and are shown to have contractile properties due to their cytoplasmic extensions that wrap around the endothelial cells lining the capillaries and venules throughout the body [1,2,3,4]. While pericytes do not possess any unique biomarkers, they can be identified by their expression of the platelet-derived growth factor receptor-beta (PDGFRb) and of the neural/glial antigen 2 (NG2 proteoglycan) a co-receptor for PDGF. The retina and the brain are known to have the highest density of vascular pericytes in the body [5]. Pericytes differ in origin, morphology, and function, depending on their organ-derived vascular bed. They are located abluminal to microvascular endothelial cells with which they share a common basement membrane [6,7]. The human blood–brain barrier (BBB) consists of brain microvascular endothelial cells, brain vascular pericytes, and astrocytes; together they are known as the neurovascular unit (NVU). Pericytes of the BBB play an essential role in a range of microvascular functions, including angiogenesis, vascular remodeling, regression, and stabilization, as well as generation and maintenance of the BBB [8,9,10,11]. The inner blood–retinal barrier (IBRB) consists of retinal microvascular endothelial cells covered with tightly associated pericytes and Müller cells; together, they make up the retinal vascular unit (RVU) [12]. Retinal and brain pericytes have similar functions and both require regulation of PDGFRb signaling for proper pericyte recruitment and adequate barrier formation [13,14]. A blood–placental barrier model consisting of trophoblasts and placental pericytes has been established [15]. This model is characterized by molecular permeability, transepithelial electrical resistance, and cell-cell-contact markers [15]. Placental pericytes are essential for endothelial cell proliferation as well as for placental microvasculature stability and integrity but have largely been ignored in placenta biology. Pericytes are also critical for placental vascular development and angiogenesis [15,16,17,18,19]. Mesangial cells are considered specialized pericytes of the renal glomerulus [20,21,22,23,24,25,26]. The glomerular vascular unit (GVU) consists of glomerular endothelial cells, podocytes, and mesangial cells [20]. The molecular crosstalk between mesangial cells, podocytes, and glomerular endothelial cells is essential for glomerular filtration. Mesangial cells form a supporting framework that maintains the structural integrity of the glomerular tuft that includes the glomerular capillaries [21]. Mesangial cells affect glomerular hemodynamics by altering glomerular vascular resistance [21].

A literature search on PubMed for “cytomegalovirus and pericytes” yielded 13 publications, about half of which are relevant to human diseases. The studies described in this review reported evidence of complete lytic replication of human cytomegalovirus (HCMV) in the brain, retinal, placental, and glomerular (mesangial cells), pericytes populations that resulted in the induction of pro-inflammatory cytokines that likely contributed to vascular inflammation. These studies relied mainly on in vitro data obtained from experiments using primary cells at low passage and infection at low multiplicity. Results from these studies support the notion that all pericytes populations, regardless of origin, are permissive for HCMV infection, and that loss of HCMV immune surveillance or intermittent viral shedding over time could contribute to pericyte loss or dysfunction, microvascular instability, and subclinical progressive vascular diseases.

## 2. CMV Infection of Brain Vascular Pericytes: Implications for HCMV-Associated CNS Vascular Disease

**Figure 1:**
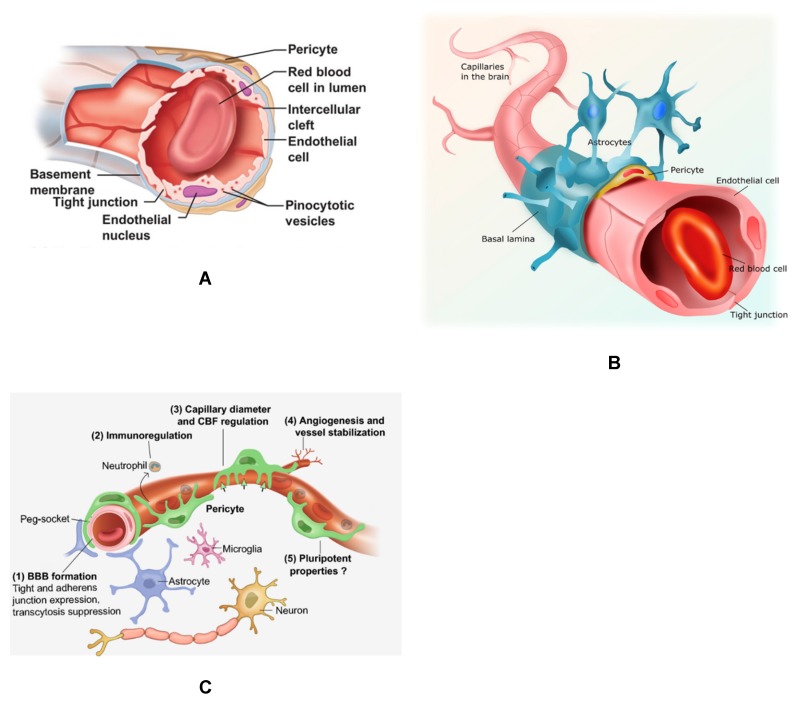
Brain capillaries featuring pericytes. (**A**) Cross-section of a brain capillary illustrating the capillary basement membrane, as well as the endothelial cells and brain pericytes abluminal to endothelial cells sharing a contiguous basement membrane. (**B**). The neurovascular unit (NVU) of the blood–brain barrier (BBB) featuring capillary endothelial cells with tight junction, pericytes and astrocytes. (**C**) The NVU and other brain parenchymal cells, including neurons and microglia. Important functions of pericytes are also shown listed 1–5.

The restrictive BBB interacts with the peripheral circulation and the central nervous system (CNS) allowing crosstalk and signaling among cellular components of the NVU in the CNS to prevent bloodborne pathogens from harming the brain. Pericytes of the BBB are the most studied among pericytes populations and are featured in this review (Figure 1). This barrier is an elaborate network of tight junctions (TJ) between capillary endothelial cells that lack fenestrae and have a reduced capacity for pinocytosis [27,28] (Figure 1A). The tight junctions of the capillary endothelium are supported by astrocytic endfeet and pericytes (Figure 1B). Brain pericytes have been shown to enhance TJ barrier function, stimulate expression of TJ proteins, and reduce the paracellular permeability of the capillary endothelium [29,30,31,32]. Brain pericytes actively communicate with other cells of the neurovasculature, including endothelial cells, astrocytes, and other parenchymal cells such as neurons and microglia (Figure 1B,C). Pericytes are essential for the BBB formation through tight junction and adherens junction formation, immunoregulation, angiogenesis, and regulation of capillary diameter and cerebral blood flow (CBF). These pericytes are pluripotent (Figure 1C).

**Figure 2:**
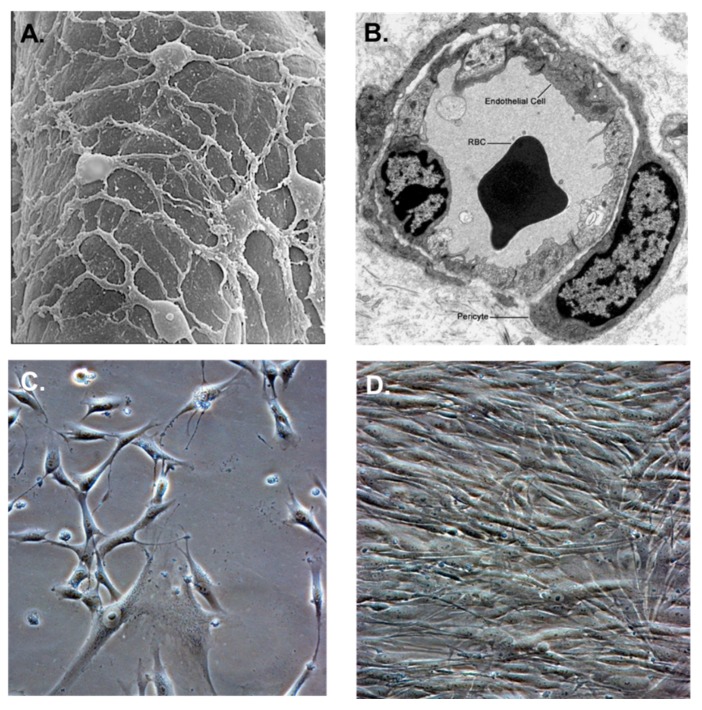
Pericyte morphology and cultivation. (**A**) Transmission electron micrograph of the abluminal surface of a rat brain capillary showing the arrangement of pericytes with long cytoplasmic extensions. (**B**) Cross-section of a rat brain capillary showing microvascular endothelial cells and pericytes. (**C**) Subconfluent culture of normal primary human brain pericytes with long cytoplasmic extensions. (**D**) Confluent culture of normal primary human brain pericytes. Images (**A**) and (**B**) (modified with permission from Pearson Education Inc. (unpublished data). Phase images for (**C**) and (**D**) were taken on a Nikon TE2000S microscope mounted with a charge-coupled device (CCD) camera at ×200 magnification.

Pericytes are contractile and migratory stem cells [33,34] that surround capillaries (Figure 2A) and are themselves surrounded by a basal lamina (Figure 2B). They also contribute to the deposition of the basal lamina during vascular development and angiogenesis [35]. Pericytes not only regulate blood flow, vascular permeability, and leukocyte recruitment but also contribute to maladaptive tissue responses, such as fibrosis [36]. Pericyte cultivated in vitro exhibit long cytoplasmic extensions that are more pronounced in subconfluent cultures (Figure 2C), but they resemble fibroblasts when confluent in cell culture (Figure 2D) [31].

Although pericytes are critical to the development and function of the BBB, their role in HCMV infection and dissemination has largely been ignored in the scientific literature [37]. Rather, to date, astrocytes and brain microvascular endothelial cells (BMVEC) cells have been implicated in supporting HCMV dissemination at the BBB [38].

Using a clinical isolate of HCMV (SBCMV) to infect pericyte cultures, Alcendor et al. showed evidence of cell lysis and typical cytomegalic cytopathology. The authors confirmed HCMV infection by detecting the expression of the major immediate early MIE and late virion protein pp65, and by the presence of HCMV mRNA in cultured cells [11]. They also found that brain pericytes were fully permissive for CMV lytic replication after 96 h in culture compared to primary human astrocytes or primary human brain microvascular endothelial cells (BMVEC) [11]. However, they showed that temporal transcriptional expression of the pp65 gene after SBCMV infection was lower than that seen with infection by the HCMV Towne laboratory-adapted strain. Proinflammatory cytokines CXCL8/IL-8, CXCL11/ITAC, and CCL5/RANTES (regulated upon activation of normal T cell expressed and secreted) were upregulated in SBCMV infected brain pericytes, as were tumor necrosis factor-alpha (TNF-α), interleukin-1 beta (IL-1β), and interleukin-6 (IL-6) [11]. Pericytes exposed to SBCMV elicited higher levels of IL-6 compared to both mock-infected cells and cells exposed to a heat-inactivated virus. Induction of IL-6 was observed in SBCMV infected cell supernatants at 24 h after infection, but the induction of TNF-α was absent [11]. Their examination of archival tissue from a patient coinfected with HCMV and HIV using dual-label immunohistochemistry also uncovered evidence of HCMV infection in pericytes, as indicated by NG2 proteoglycan staining [11]. The presence of HCMV lytic infection in primary human brain pericytes suggests that pericytes likely contribute to both virus dissemination in the CNS and neuroinflammation.

The data from this study indicate that brain pericytes are more permissive for CMV lytic replication compared to BMVEC or astrocytes and that pericytes could serve as amplification reservoirs for HCMV. Finally, pericyte exposure to HCMV can induce a proinflammatory cascade that likely contributes to neuroinflammation.

## 3. Cytomegalovirus and Retinal Pericytes: Implications in Ocular Disease

Retinal pericytes play an essential role in maintaining retinal vascular and endothelial cell proliferation [39,40,41]. Defined mechanisms of HCMV ocular pathogenesis that involves retinal pericytes are unknown. This lack of knowledge represents a major gap in understanding HCMV- associated ocular diseases. Congenital HCMV infection is the major cause of birth defects affecting approximately 40,000 children (0.2 to 2% of all live births) born in the United States each year [42,43,44,45]. CNS abnormalities in newborns include vision loss, mental retardation, motor deficits, seizures, and sensorineural hearing loss (SNHL) [36,46]. Retinitis due to HCMV infection can also result in blindness and is the most prevalent ocular disease in individuals with HIV/AIDS [47,48]. Other retinal pathologies associated with HCMV infection include age-related macular degeneration (AMD), which is the leading cause of irreversible central vision loss and legal blindness in older adults worldwide [49,50]. An association was previously observed between the high HCMV IgG titers and the neovascular form of AMD. Therefore, it has been proposed that chronic HCMV infection could be a novel risk factor for the progression from the dry form of AMD to the neovascular or wet form of AMD [49].

HCMV infection of retinal pericytes is a lytic infection that would result in pericyte loss at proximal sites within the IBRB [12]. This may affect retinal neurovascular permeability as well as an increase in retinal inflammation and angiogenesis [12]. Previous studies by Alcendor et al. and Wilkerson et al. showed vascular pericytes to be the most permissive cell type to HCMV infection within the BBB and the IBRB [11,16]. These findings suggest that vascular pericytes may serve as amplification reservoir for HCMV dissemination within these vascular beds.

Retinal pericytes expressed the biomarker neuron-glial antigen 2. Antigenic expression profiles for several cytoskeletal, cell adhesion and inflammatory proteins were shared by both retinal and brain pericytes. Wilkerson et al. demonstrated that HCMV-infected pericytes showed cytomegalic cytopathology and expressed mRNAs encoding major immediate early (MIE) proteins and the HCMV phosphorylated envelop protein 65 [12]. Their quantitative real-time polymerase chain reaction (qRT-PCR) analysis confirmed full lytic replication of HCMV in retinal pericytes. They presented Luminex analysis of supernatants from SBCMV-infected retinal pericytes that revealed increased levels of macrophage inflammatory protein-1α (MIP-1α), beta-2 microglobulin (β2-m), and matrix metalloproteinase-3 and -9 (MMP3/9), as well as the lower levels of IL-6 and IL-8 compared to uninfected controls. At 24 h post infection, these pericytes expressed higher levels of IL-8, TIMP-1 (tissue inhibitor of metalloproteinase-1), and RANTES, but lower levels of MMP9 [12]. A time course analysis showed that both the brain and the retinal pericytes were more permissive for HCMV infection than other cellular components of the BBB and IBRB. Using a Tricell culture model of the IBRB consisting of retinal endothelial, retinal pericytes, Müller cells, the authors found that retinal pericytes were most permissive for SBCMV infection [12]. SBCMV infection of this IBRB Tricell mixture for 96 h resulted in increased levels of IL-6, MMP9, and stem cell factor with a concomitant decrease in granulocyte-macrophage colony-stimulating factor and TNF-α [12]. In retinal pericytes, HCMV induces proinflammatory and angiogenic cytokines. Therefore, pericytes in the IBRB likely serve as an amplification reservoir which contributes to retinal inflammation and angiogenesis.

## 4. Placental Pericytes and Cytomegalovirus Infection: Implication for CMV-Induced Congenital Diseases

Congenital HCMV infection can result in several abnormalities including vision loss, mental retardation, motor deficits, seizures, and hearing loss [43,51,52,53,54]. Forty percent of mothers with primary HCMV infection during gestation transmit the virus to their babies [43,55]. Moreover, 58% of transplacental transmission of HCMV occur in women who are seropositive with non-primary maternal infections [56]. Annually, 0.5% to 3.0% of all newborns in the US are infected with HCMV [57]. HCMV causes not only life-threatening diseases in immunocompromised individuals [58,59], but also HCMV-associated pathologies that lead to long-term health risks. There will likely be higher incidences of HCMV congenital disease in a population with lower socioeconomic status and higher HCMV seroprevalence that will vary among global populations. Therefore, the occurrence of HCMV infection represents an important health disparity in underserved communities. Higher infection rates are observed among non-Hispanic Blacks and Mexican Americans than among non-Hispanic Whites [60]. HCMV infection disrupts normal placental function and development; hence congenital HCMV should be considered as an underlying cause of intrauterine growth restriction (IUGR) [61].

Placental pericytes are essential for endothelial cell proliferation as well as placental microvasculature stability and integrity but have largely been ignored in placenta biology [62]. Due to their pluripotent nature, pericytes are critical for placental vascular development and angiogenesis [19,63]. A study by Aronoff et al. examined the secretion profile of placental pericytes alone at 24 h post-exposure to SBCMV revealed increased expression of monocyte chemotactic protein-1 (MCP-1) [16]. Hamilton et al. also observed an increased MCP-1 expression in ex vivo placental histocultures infected with laboratory and clinical HCMV strains [64]. These results suggest that HCMV infection alters the placental microenvironment and MCP-1 initiates placental and fetal injury [65]. Aronoff et al. observed increased levels of VEGF in supernatants of placental pericytes exposed to SBCMV [16]. A study by Ahmad et al. revealed increased levels of soluble VEGF/VEGF receptor-1 in supernatants from preeclamptic placental explants, indicating the involvement of placental VEGF/VEGF receptor-1 in the inhibition of angiogenesis in preeclampsia [65]. Aronoff et al. also observed a marginal increase in RANTES levels exclusively in pericytes exposed to HCMV. Similarly, Wilkerson et al. showed increased RANTES/CCL5 expression in brain vascular pericytes exposed to HCMV [12]. They also showed that IL-6 levels in SBCMV-infected and heat-inactivated virus-infected pericytes were lower compared to that in mock-infected pericytes. Other investigators found that IL-6 levels were suppressed in human fibroblasts undergoing active infection, mediated in part by HCMV IE2 proteins and post-transcriptional destabilization of IL-6 mRNA [66]. HCMV infection of placental pericytes is a lytic infection that can cause pericyte loss at proximal sites within the placental vasculature. This may affect placental vascular permeability and increase in placental inflammation and angiogenesis. Moreover, impairment of tissue perfusion and microcirculatory abnormalities are also likely. HCMV placental pathogenesis models that include placental pericytes have not been reported. How signaling occurs among placental pericytes, cytotrophoblast, and villous fibroblasts are largely unknown. Therefore, additional studies investigating the infectivity of human placental pericytes by HCMV and their potential role in viral dissemination in placental tissue as well as their implication in HCMV-associated congenital diseases are essential.

## 5. Mesangial Cells (Specialized Renal Pericytes) and Cytomegalovirus Infectivity: Implications for Kidney Disease

HCMV is the most threatening viral pathogen after kidney transplantation [67,68]. HCMV is a leading cause of post-transplant morbidity and mortality [69]. Clinical manifestations of HCMV infection include myelosuppression, fever, retinitis, pneumonia, colitis, and hepatitis [70]. HCMV infection of kidney allograft transplant patients can result in reduced graft and patient survival as well as increased risk of graft rejection and susceptibility to other opportunistic infections [71,72,73,74]. Without HCMV prophylaxis, 40% to 100% of all kidney transplant recipients (KTRs) will become infected with HCMV, and up to 67% will develop HCMV-associated clinical disease. With HCMV prophylaxis, the disease incidence is reduced to no more than 37% [75]. Risk factors for HCMV-associated disease in KTRs that most often occur in the first 100 days post-transplant include kidney-pancreas transplantation, type of immunosuppressive drugs used, serostatus of the donor and recipient, the presence or the absence of acute rejection, donor age of >60 years, and impaired graft function [75,76,77]. There have been case reports of mesangial sclerosis in HCMV infected patients with congenital nephrotic syndrome. Renal biopsies of these patients reveal diffused mesangial sclerosis and cytomegalic inclusion in both tubular cells and glomeruli [78]. Ortmanns et al. detected HCMV infection of mesangial cells in patients with IgA nephropathy and showed that treatment with ganciclovir resulted in remission [79]. It has been reported that primary human mesangial cells as well as human glomerular epithelial, tubular epithelial, and endothelial cells are permissive for HCMV infection [80,81,82]. However, these studies were limited primarily to a comparative analysis of viral infectivity. To date, how mesangial cells contribute to HCMV infection in the glomerulus is poorly understood. The molecular crosstalk between mesangial cells, podocytes, and glomerular endothelial cells, which together are known as the GVU, during HCMV infection is also poorly understood. In a study by Popik et al., the authors examined the GVU for HCMV infectivity, replication kinetics and temporal cytokines expression profiles in a glomerulus tricell culture model.

Intraglomerular mesangial cells are specialized pericytes located among the glomerular capillaries within a renal corpuscle of a kidney [22,23,24,25,26,35]. Mesangial cells are pericytes of the kidney that are abluminal to glomerular capillaries and central to the glomerular tufts between the capillary loops. These cells synthesize the mesangial cell matrix and regulate glomerular hemodynamics via cell contraction and release of vasoactive hormones. Mesangial cells have morphological characteristics that are similar to those of other pericytes; they also express pericytes antigenic biomarkers NG-2P, vimentin, CD68, and fibronectin [19,26]. Mesangial cells, like pericytes of the BBB and the IBRB previously reported by Alcendor et al. are permissive for infection by both laboratory-adapted and clinical HCMV strains; they are also the most permissive target cells in both the neurovascular units of the brain and the retina [19,26]. The angiogenic and proinflammatory expression profile we observed in the tricell GVU model would result in glomerular inflammation leading to podocyte injury, mesangial cell activation, subsequently contributing to matrix deposition, and glomerulosclerosis [20]. The proinflammatory and angiogenic cytokines induced by HCMV infection of mesangial and GVU cells have been shown to promote reactivation of HCMV from latency [83]. MIP-1α, MCP-1, and IL-8 are chemokines crucial to the attraction and activation of granulocytes, T cells, and monocytes, all of which have been implicated in the dissemination of HCMV and can promote HCMV pathology in renal transplant patients [84,85].

**Figure 3:**
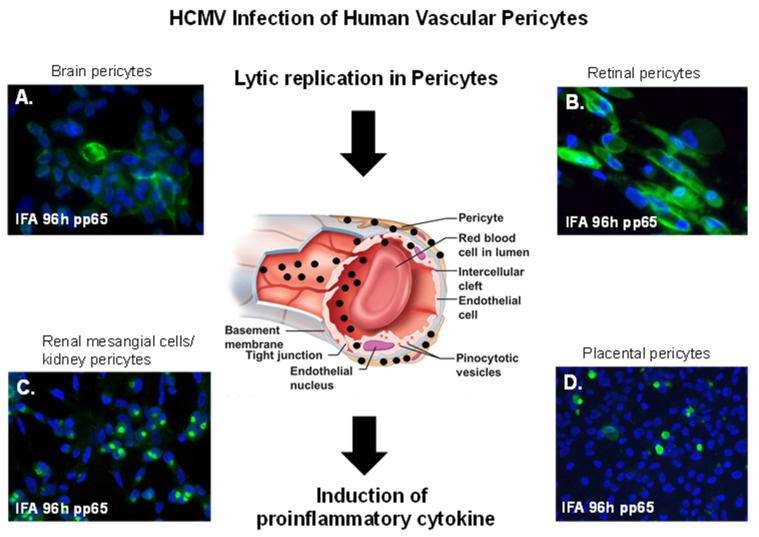
Vascular pericytes and HCMV infectivity. HCMV lytic infection of human pericytes. Black arrows point to a cross-section of a capillary showing HCMV dissemination in vasculature with virus infection concentrated in pericytes leading to the induction proinflammatory cytokines. (**A**) Immunofluorescent staining of primary human brain pericytes in with SBCMV 96 h after infection using a mouse monoclonal antibody to the HCMV pp65 tegument protein. (**B**) Immunostaining of primary human retinal pericytes infected with SBCMV at 96 h stained with the HCMV pp65 antibody. (**C**) Immunostaining of primary human renal mesangial cells infected with HCMV at 96 h and stained with the HCMV pp65 antibody. (**D**) Immunostaining of primary human placental pericytes infected with SBCMV at 96 h stained with the HCMV pp65 antibody. All images were taken on a Nikon TE2000S microscope mounted with a CCD camera at ×200 magnification. For fluorescent images, 4′,6-diamidino-2-phenylindole (DAPI) was used to stain the nuclei (blue).

Published reports on HCMV infection in the BBB, the IBRB, and the blood–placental barrier support the notion that pericytes abluminal to endothelial cell of these different microvascular compartments, including brain pericytes, retinal pericytes, placental pericytes, and glomerular pericytes (mesangial cells), were all found to be the most permissive cell type in the vascular bed for HCMV infection [11,12,16,20] (Figure 3). Human retinal pericytes were also found to be most permissive for Zika virus when compared to retinal endothelial cells and Müller cell [28]. These findings support the notion that all vascular pericytes are permissive for HCMV infection regardless of their source, and that all pericytes support the greatest viral burden, and serve as amplification reservoirs for HCMV infection and dissemination in their respective vascular beds. We previously observed that among cellular components of the GVU, glomerular endothelial cells supported higher replication levels of laboratory-adapted strains of HCMV when compared to mesangial cells, but that mesangial cells supported higher replication levels of clinical HCMV strains.

## 6. HCMV Infection as A Risk Factor for Vascular Disease

HCMV infection has been shown to be a risk factor for the development of atherosclerosis [86]. A meta-analysis of data retrieved from electronic databases PubMed, Embase, and CNKI that involved 30 studies, 3328 cases, and 2090 controls showed that HCMV infection is significantly associated with an increased risk for atherosclerosis [86]. A meta-analysis conducted by Lv et al. involving 68 studies, from 24 countries (12027 cases and 15386 controls) suggests that HCMV infection is associated with an increased risk for vascular diseases [87]. The study found that people exposed to HCMV infection had a higher risk for vascular diseases (OR 1.70 [95% CI 1.43–2.03]) [87]. In addition, a study by Huang et al. involving 200 patients diagnosed with stroke and 200 controls found a correlation between HCMV and stroke [82]. HCMV seropositivity was higher in the stroke patients than in controls (55.0% vs. 23.5%; *p* < 0.0001). Hung et al. also observed that the presence of HCMV DNA increased the risk of stroke [88]. The mechanisms associated with HCMV infection and the increased risks for the development of vascular diseases requires further investigation.

## 7. Conclusions

Primary human brain pericyte, human retinal pericytes, placental pericytes, and glomerular mesangial cells are all permissive for both laboratory-adapted and clinical HCMV strains; all were resulting in the induction of proinflammatory cytokines [11,12,16,20] (Figure 3). HCMV infection is ubiquitous in the general population, and healthy individuals will shed virus intermittently over a life span. HCMV infection that results in clinical diseases usually occurs in individuals who are immune compromised, such as transplant patients and individuals with HIV/AIDS who are not receiving antiretroviral therapy. In the elderly, HCMV-associated clinical disease can occur due to waning immunosurveillance. Taken together, these observations indicate that patients with subclinical or chronic HCMV infection could be at higher risk for developing vascular diseases such as diabetic retinopathy and age-related macular degeneration (AMD), as well as the development and progression of atherosclerosis where pericyte loss, pericyte related angiogenic dysfunction, or induction of smooth cell proliferation by HCMV infection of pericyte occurs respectively.

Model systems that closely mimic the NVU of the IBRB would improve our understanding of the development of retinopathies associated with loss of pericytes and IBRB integrity. Understanding the underlying role of HCMV in vascular disease development and progression could provide new information for developing novel therapeutic approaches for stroke and other disorders with a vascular component, including Alzheimer’s disease. Thus, therapeutic modalities designed to specifically protect pericytes from infection might include the use of biospecific molecules such as a dual-specific antibody that can interact with pericytes and neutralize HCMV concurrently.
